# Impaired Neurosphere Formation as a Functional Readout of Aberrant EGFR Signaling Kinetics in Olfactory Neural Progenitors From Patients With Schizophrenia

**DOI:** 10.1002/cbin.70181

**Published:** 2026-06-29

**Authors:** Tommaso Toffanin, Mario Angelo Pagano, Carlo Idotta, Mauro Salvi, Valentina Bosello‐Travain, Maria Lina Massimino, Roberto Saetti, Marina Silvestrini, Leonardo Meneghetti, Stefano Piazza, Maria Giulia Nanni, Rosangela Caruso, Maria Ferrara, Chiara Montemitro, Luigi Zerbinati, Martino Belvederi Murri, Luigi Grassi, Anna Maria Brunati

**Affiliations:** ^1^ Department of Neuroscience and Rehabilitation, Institute of Psychiatry University of Ferrara Ferrara Italy; ^2^ Metis ETS San Martino di Lupari Italy; ^3^ Department of Biomedical Sciences University of Padova Padova Italy; ^4^ Department of Molecular Medicine University of Padova Padova Italy; ^5^ Neuroscience Institute, Section of Padova National Research Council (CNR) Padova Italy; ^6^ Department of Otolaryngology San Bortolo Hospital Vicenza Italy; ^7^ Department of Mental Health Vicenza Italy

**Keywords:** epidermal growth factor receptor, neurospheres, olfactory neuroepithelium, schizophrenia

## Abstract

The Epidermal Growth Factor Receptor (EGFR) is a key regulator of neurodevelopment, controlling the proliferation, differentiation, and self‐renewal of neural stem and progenitor cells. Dysregulated EGFR signaling has been implicated in schizophrenia, a neurodevelopmental disorder associated with abnormal cortical maturation. We investigated EGFR‐dependent signaling in neural stem/progenitor cells derived from olfactory neuroepithelium (hereafter referred to as Olfactory Neural Stem/Progenitor Cells, ONSPCs) collected from individuals with no prior history of psychiatric disorders (hereafter referred to as healthy controls, HC) and patients diagnosed with schizophrenia (schizophrenic, SZ), an ex vivo human model that retains disease‐related molecular signatures. Both HC‐ and SZ‐derived ONSPCs formed neurospheres under proliferative conditions in the presence of the growth factors Epidermal Growth Factor (EGF) and Fibroblast Growth Factor (FGF), confirming their neural progenitor identity. However, under EGF‐exclusive conditions, HC‐derived ONSPCs generated compact, well‐organized neurospheres, whereas SZ‐derived ONSPCs produced sparse, irregular aggregates, indicating impaired responsiveness to EGF. Despite comparable EGFR protein levels between groups, EGF stimulation revealed distinct dynamics of downstream EGFR effectors. In HC‐derived ONSPCs, EGFR activation was initially modest but sustained over time, whereas in SZ‐derived cells it was more rapid and transiently stronger, followed by early signal decay. In particular, Akt and Src, which are implicated in signaling pathways driving proliferation and self‐renewal, displayed activation dynamics paralleling those of EGFR in each group. These findings are consistent with dysregulation of the magnitude and temporal dynamics of EGFR–dependent signaling in SZ‐derived ONSPCs, associated with impaired neurosphere formation under EGF‐exclusive conditions and suggestive of reduced self‐renewal capacity. Although limited by the modest sample size, the semiquantitative nature of Western blot analyses, and the potential effect of medication exposure, this study supports the relevance of patient‐derived ONSPCs as a physiologically meaningful platform for investigating neurodevelopmental mechanisms underlying schizophrenia.

**Abbreviations**
AKT: AK Thymoma (aka Protein Kinase B)
ANOVA: analysis of variance
DMEM/F‐12: Dulbecco's Modified Eagle/F‐12 medium
DSM‐5: diagnostic and statistical manual of mental disorders, Fifth Edition
ECL: enhanced chemiluminescence
EGF: epidermal growth factor
EGFR: epidermal growth factor receptor
ERK: extracellular signal‐regulated kinase
FGF: fibroblast growth factor
HC: healthy control
JAK: Janus Kinase
MAPK: mitogen‐activated protein Kinase
MEK: mitogen‐activated Erk Kinase (aka Mitogen‐activated kinase kinase)
mTOR: mammalian target of rapamycin
ONSPCs: olfactory neural stem/progenitor cells
PI3K: phosphoinositide 3‐kinase
PKC: protein kinase C
PLCγ: Phospholipase Cγ
RAF: rapidly accelerated fibrosarcoma
RAS: rat sarcoma
SD: standard deviation
SDS‐PAGE: sodium dodecyl Sulfate‐Polyacrylamide gel electrophoresis
SGZ: Subgranular Zone
Src: sarcoma kinase
STAT: signal transducer and activator of transcription
SVZ: subventricular zone
SZ: schizophrenic
TGF‐α: transforming growth factor‐α
WB: western blot

## 1 Introduction

The Epidermal Growth Factor Receptor (EGFR/ErbB1/HER1) is a master regulator of tissue homeostasis, governing development, repair, and stem cell maintenance across epithelial, mesenchymal, and stromal lineages (Chen et al. 2016; Hu et al. 2010; Yu et al. 2017). Structurally, it is a transmembrane tyrosine kinase of the ErbB family, which also includes ErbB2/HER2, ErbB3/HER3, and ErbB4/HER4 (Lemmon et al. 2014). EGFR activation by its primary ligands, Epidermal Growth Factor (EGF) and Transforming Growth Factor‐α (TGF‐α), initiates key downstream signaling cascades, including RAS/RAF/MEK/ERK (proliferation, transcription), PI3K/AKT/mTOR (growth, survival), JAK/STAT, and PLCγ/PKC (differentiation, cytoskeletal dynamics, calcium signaling), collectively orchestrating a wide spectrum of cellular processes (Lemmon and Schlessinger 2010; Purba et al. 2017).

The pathogenic role of EGFR signaling dysregulation, long recognized as a hallmark of diverse cancers (Levantini et al. 2022) and more recently implicated in chronic inflammation and organ fibrosis (Pastore et al. 2008; Ezaddoustdar et al. 2025), has spurred the development of multiple EGFR‐targeted treatments, including tyrosine kinase inhibitors (e.g., gefitinib, erlotinib, afatinib, osimertinib) and monoclonal antibodies (e.g., cetuximab, panitumumab) (Gao et al. 2025). EGFR also plays a central role during neurodevelopment, as evidenced by its expression in neural stem cells and neural progenitor cells (Burrows et al. 1997), regulating the balance between symmetric and asymmetric divisions of radial glial cells—primary progenitors of the developing cortex—thereby controlling the expansion of the progenitor pool and influencing brain size and architecture (Lillien and Raphael 2000; Sun et al. 2005). Within postnatal and adult neurogenic niches—the subventricular zone (SVZ) of the lateral ventricles and the subgranular zone (SGZ) of the hippocampal dentate gyrus—EGFR signaling maintains neural stem/progenitor cells in a self‐renewing, undifferentiated state by promoting proliferation and suppressing differentiation (Doetsch et al. 2002; Aguirre et al. 2010). Conversely, studies in knockout mouse models demonstrate that EGFR dysfunction leads to postnatal neurodegeneration, glial deficits, and altered migration, whereas gliosis and abnormal neural patterning characterize mice overexpressing EGFR (Sibilia and Wagner 1995; Wagner et al. 2006).

A growing body of evidence supports a neurodevelopmental model of psychiatric disorders, in which genetic and environmental insults perturb signaling pathways critical for brain maturation, producing lasting structural and functional deficits (Rapoport et al. 2005). In schizophrenia specifically, neurodevelopmental alterations have been ascribed to aberrant EGF/ErbB signaling, with reduced cortical EGF levels and increased EGFR expression documented in post‐mortem tissue (Futamura et al. 2002), as well as downregulation of the EGFR‐PI3K‐AKT pathway in the prefrontal cortex (Pantazopoulos et al. 2021). In rodent models, perinatal EGF exposure induces behavioral and neurochemical alterations resembling schizophrenia, reversible via EGFR inhibition (Namba and Nawa 2020).

To investigate these mechanisms in a physiologically relevant human model, we employed olfactory neural stem/progenitor cells (ONSPCs), an ex vivo system derived from the nasal neurogenic niche that recapitulates key features of the SVZ and SGZ and retains disease‐related molecular and cellular signatures (Benítez‐King et al. 2011; Mackay‐Sim 2012). The capacity of this model to detect pathophysiologically meaningful alterations in schizophrenia is supported by seminal studies reporting dysregulated proliferation and cell cycle dynamics in olfactory neuroepithelium‐derived cultures from individuals with schizophrenia (Féron et al. 1999; McCurdy et al. 2006; Fan et al. 2012), and more recently corroborated by evidence from our group documenting molecular and cellular correlates of the disorder in ONSPCs (Idotta et al. 2024).

Building on this evidence, we sought to characterize EGFR‐dependent signaling in ONSPCs derived from individuals with no prior history of psychiatric disorders (healthy controls, HC) and patients diagnosed with schizophrenia (schizophrenic, SZ), using neurosphere formation and phosphorylation of key downstream effectors as functional readouts. Our results show that HC‐ and SZ‐derived ONSPCs respond markedly differently to EGF stimulation, exhibiting distinct signaling dynamics consistent with a role for EGFR dysregulation in the pathophysiology of schizophrenia.

## 2 Materials and Methods

### 2.1 Ethics Approval, Patient Recruitment, and Consent to Participate

This study was conducted in compliance with the ethical principles outlined in the Declaration of Helsinki and was approved by the Vicenza Ethical Review Committee (Protocol No. 1022/23). All participants provided written informed consent prior to enrollment. Six outpatients diagnosed with schizophrenia, previously hospitalized at the Department of Mental Health, Vicenza Hospital, and stabilized on antipsychotic treatment after discharge, and six individuals with no lifetime history of psychiatric disorders, as assessed through clinical interview and medical record review, were enrolled in the present study.

Patients, aged 18–65, met the criteria of the Diagnostic and Statistical Manual of Mental Disorders, Fifth Edition (DSM‐5), for schizophrenia. Exclusion criteria for all participants included a comorbid DSM‐5 diagnosis of substance use or addictive disorder in the preceding 12 months, any other mental disorder, neurological disease, or a head injury with documented loss of consciousness exceeding 5 min.

### 2.2 Isolation, Propagation, and Storage of ONSPCs

ONSPCs were collected by brushing the epithelial surface of the middle turbinate of both nostrils with a dedicated brush, as previously described (Idotta et al. 2024). Disinfection with 0.5% chlorhexidine in the nasal vestibule was performed when required (nasal vestibulitis or marked nasal discharge). The specimens were harvested in a 15 mL tube filled with 6 mL Dulbecco's Modified Eagle/F‐12 medium (DMEM/F‐12, Merck) supplemented with 10% fetal bovine serum (Merck), 4 mM l‐glutamine (Merck), and 100 μg/mL Primocin™ (Invivogen) (hereafter complete DMEM/F‐12 medium), and subsequently transferred into T25 flasks. Upon reaching confluence, cells were trypsin‐dissociated and transferred into T75 flasks for further expansion to 80% confluence. If not used for further experiments, ONSPCs were cryopreserved at −80°C in ENStem‐A Neural Freezing Medium (Merck).

### 2.3 Formation of Neurospheres in Complete Proliferation Medium and EGF‐Exclusive Medium

Cryopreserved ONSPCs from HC donors and SZ patients were rapidly thawed in a 37°C water bath for 30 s, resuspended in complete DMEM/F12 medium, seeded into T75 flasks, and incubated at 37°C under 5% CO_2_. Upon reaching 80% confluence, ONSPCs were detached by trypsinization, collected in a 15 mL tube, and centrifuged at 200 × g for 3 min, and washed twice in NS‐A Proliferation Medium (StemCell^TM^ Technologies) devoid of growth factors. ONSPCs were then resuspended in NS‐A Proliferation Medium supplemented with 20 ng/mL EGF, 10 ng/mL bFGF, and 0.00004% heparin (StemCell^TM^ Technologies) (hereafter referred to as complete NS‐A Proliferation Medium) and incubated at 37°C under 5% CO_2_ for 48 h to promote neurosphere formation. The neurospheres were transferred to, and allowed to sediment, in 15‐mL tubes for 20 min, and then treated with trypsin at 37°C under 5% CO_2_ for 10 min and triturated to obtain single‐cell suspensions. For quantitative comparison of neurosphere‐forming capacity between groups, ONSPCs from the neurosphere‐derived single‐cell suspension were seeded into 12‐well plates (6 × 10^5^ cells per well) in serum‐free DMEM/F12 medium supplemented with 20 ng/mL EGF and incubated for 48 h.

### 2.4 Microscopy and Image Acquisition

ONSPCs and neurospheres were visualized using an Axio Vert.A1 FL‐LED Inverted Microscope equipped with an AxioCam camera system and captured via the Zen 3.2 software (Carl Zeiss), and subsequently analyzed using ImageJ software (NIH, Bethesda, MD, USA).

### 2.5 Cell Counting

Prior to all experimental procedures, viable ONSPCs were quantified by Trypan blue exclusion assay with a Bürker hemocytometer. Neurosphere number was subsequently determined by analyzing microscopy images acquired as described above, counting six randomly selected, non‐intersecting fields per well and extrapolating the total to the entire well surface area, while neurosphere diameter was measured using a calibrated scale bar. Only neurospheres measuring at least 30 μm in diameter were included in the analysis.

### 2.6 Western Blot Analysis

ONSPCs were seeded in 12‐well plates (6 × 10^5^ cells per well) and, after the indicated treatments and incubation times, lysed directly in the wells. One quarter of the total lysate from each well was resolved by 10% SDS‐PAGE and transferred onto nitrocellulose membranes for Western blot (WB) analysis. After 1 h incubation with 3% bovine serum albumin at room temperature, membranes were incubated with the appropriate antibodies overnight. The primary antibodies included anti‐phosphotyrosine (Merck), anti‐p44/42 MAPK (ERK1/2), anti‐phospho‐p44/42 MAPK (ERK1/2) (Thr202/Tyr204), anti‐Akt, anti‐phospho‐Akt (Ser473), anti‐Src antibody, anti‐phospho‐Src family (Tyr416), anti‐EGF Receptor, anti‐phospho‐EGF Receptor (Tyr1068) (Cell Signaling Technology), and anti‐Neuron‐specific β‐III Tubulin (Selleckchem). Immunodetection was carried out with the ECL Western Blotting Substrate (Thermo Fisher Scientific) on the Kodak Image Station 4000 mm Pro Digital System (Eastman Kodak). Band intensity was quantified by ImageJ software (National Institutes of Health, USA).

### 2.7 Statistics

All experiments were performed on biological replicates from independent donors (*n* = 6 per group). For neurosphere assays and Western blot analyses, technical triplicates were averaged to a single donor‐level value per condition and time point; data are presented as mean ± SD. Intra‐individual reproducibility was assessed by the coefficient of variation (CV%) across technical replicates. Given the limited sample size, between‐group comparisons were performed using the two‐sided Mann–Whitney *U* test, whereas paired comparisons before and after erlotinib treatment within the same donor group were analyzed using the Wilcoxon signed‐rank test. Statistical analyses were conducted in GraphPad Prism (v. 9.5.1); *p* < 0.05 was considered statistically significant.

## 3 Results

### 3.1 Neurosphere Formation and Expansion of Neurosphere‐Derived Neural Stem/Progenitor Cells

**Figure 1:**
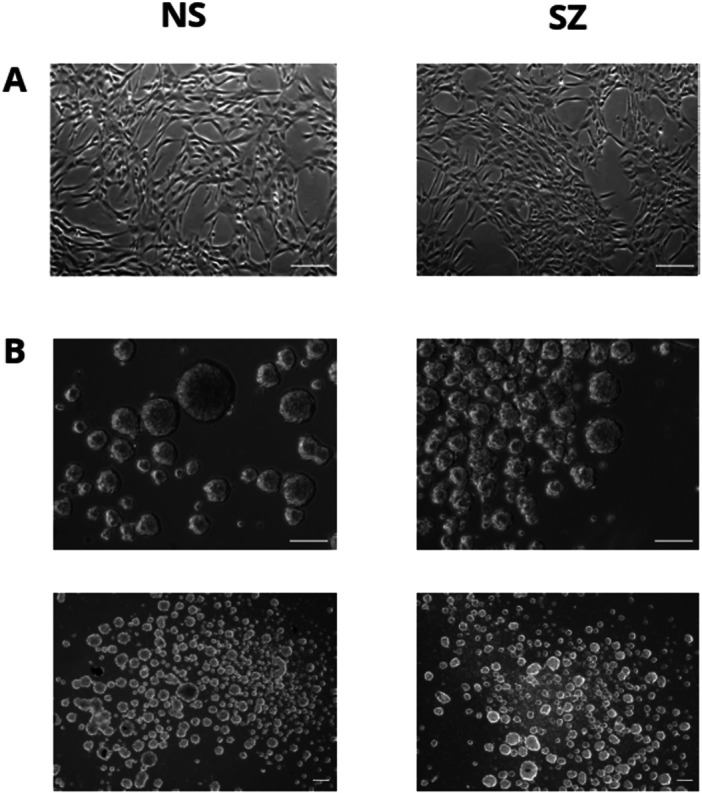
Neurosphere formation from ONSPCs. (A) ONSPCs from HC donors (left‐hand panel) and SZ patients (right‐hand panel) were expanded to 80% confluence in complete DMEM/F12 medium (images at 10× magnification). (B) Neurospheres were generated from ONSPCs from HC donors (left‐hand panel) and SZ patients (right‐hand panel), seeded at low density in NS‐A proliferation medium devoid of EGF, FGF, and heparin following trypsin dissociation of 80% confluent monolayers. The proliferation medium was subsequently supplemented with EGF, FGF, and heparin at a final concentration of 20 ng/mL, 10 ng/mL, and 0.00004%, respectively, and incubated for 48 h to generate neurospheres (images at 10× magnification, upper panel; 4× magnification, lower panel). Scale bars: 100 μm.

To confirm the neural stem/progenitor nature of ONSPCs and their capacity for self‐renewal and proliferation, ONSPCs were cultured under standard neurosphere‐forming conditions. Cryopreserved ONSPCs from 6 HC and 6 SZ donors, whose characteristics are reported in Table 1, were thawed and expanded to 80% confluence in complete DMEM/F12 medium at 37°C under 5% CO_2_ (Figure 1A). Following trypsin‐mediated detachment, ONSPCs were transferred to NS‐A Proliferation Medium supplemented with 20 ng/mL EGF, 10 ng/mL bFGF, and 0.00004% heparin, and incubated at 37°C under 5% CO_2_ to promote neurosphere formation, as elsewhere described (Vitale et al. 2017). This latter procedure mirrors established protocols for culturing neural stem cells derived from neurogenic regions such as the SVZ and the SGZ (da Silva Siqueira et al. 2021). Within approximately 48 h, numerous free‐floating neurospheres were observed in both HC and SZ cultures (Figure 1B), consistent with the proliferative response of neural stem/progenitor cells to mitogenic growth factors (Reynolds and Weiss 1992). These served as a cellular source enriched in stem/progenitor cells for subsequent experiments. The neurospheres were then recovered by sedimentation, trypsinized, and triturated to obtain a single‐cell suspension. The neurosphere‐derived cells were then incubated in complete DMEM/F12 medium and expanded to 80% confluence for downstream analyses. These results show that ONSPCs from both HC and SZ donors respond to conditions promoting neurosphere formation, established for neural stem/progenitor cells residing in established neurogenic niches (Ilaria Decimo et al. 2012), retaining comparable proliferative potential irrespective of disease status.

**Table 1:** Participant characteristics. Demographic and clinical characteristics of the six patients with schizophrenia (schizophrenic, SZ) and six participants with no prior history of psychiatric disorders (healthy controls, HC).

SZ	Age	Gender	Smoking status	Hospitalization	Duration of illness (years)	Medication	CPZ equivalents	Concomitant medications
1	35	M	Y	2	13	aripiprazole	833	
2	48	F	Y	4	20	risperidone	375	
3	60	M	N	2	37	aripiprazole, clozapine	437	
4	29	F	N	1	7	aripiprazole	833	delorazepam
5	58	M	N	3	20	risperidone	500	ramipril
6	31	F	Y	1	6	aripiprazole	556	lorazepam
Mean ± S. D.)	40.5 ± 11.7			2.17 ± 1.17	17.2 ± 11.4			
Median (range	37.5 (29–60)			2 (1–4)	16.5 (6–37)			
**HC**								
1	35	M						
2	61	M						
3	52	F						
4	40	M						
5	38	F						
6	39	F						
Mean ± S. D.	44.2 ± 10.1							
Median (range)	39.5 (38–61)							

*Note:* No significant between‐group differences were observed in mean age (Welch's two‐tailed *t*‐test: *t*(10) = −0.58, *p* = 0.576) or sex distribution (3 of 6 males in each group; *p* = 1.000). Duration of illness was significantly correlated with age (*r* = 0.943, *p* < 0.001). All patients had at least one prior psychiatric hospitalization (mean ± SD number of hospitalizations, 2.67 ± 1.63). Antipsychotic doses were converted to chlorpromazine (CPZ) equivalents using published conversion tables (Taylor et al. 2025).

### 3.2 Differential Neurosphere‐Forming Responses to EGF in HC‐ and SZ‐Derived ONSPCs

**Figure 2:**
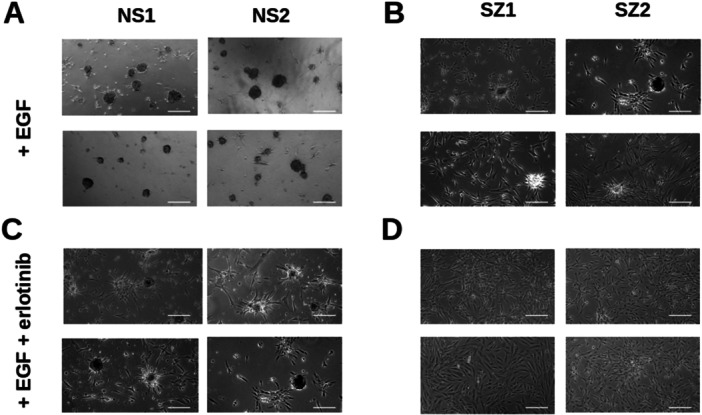
EGF‐induced formation of neurospheres from HC‐ and SZ‐derived ONSPCs under EGF‐exclusive conditions. Neurosphere‐derived ONSPCs from either HC donors (A) or SZ patients (B) were incubated in DMEM/F12 in the presence of 20 ng/mL EGF for 48 h. Alternatively, neurosphere‐derived ONSPCs from either HC donors (C) or SZ patients (D) were incubated in DMEM/F12 in the presence of the selective EGFR inhibitor erlotinib (10 μM) for 30 min prior to stimulation with 20 ng/mL EGF for 48 h. The images show ONSPCs from 2 controls and 2 patients and are representative of all the cell cultures analyzed (10× magnification). Scale bars: 100 μm.

EGF is crucial for the self‐renewal and proliferation of neural stem cells, promoting the formation of neurospheres in vitro even in the absence of other growth factors (Doetsch et al. 2002; Reynolds and Weiss 1992). Because cultivation of ONSPCs in complete NS‐A Proliferation Medium yielded numerous neurospheres irrespective of the participant group, and EGF signaling is not recapitulated by other growth factors, we sought to determine whether EGF alone, under stringent conditions, could unmask differences in ONSPC proliferative and neurosphere‐forming capacity. To this end, ONSPCs (8 × 10^4^ cells per well) from the single‐cell suspensions, obtained as described above, from both groups were seeded in 12‐well plates and cultured in serum‐free DMEM/F12 medium in the absence or presence of 20 ng/mL EGF for 48 h. As shown in Figure 2A, HC‐derived cultures formed multiple spherical cellular aggregates within 48 h. Only well‐defined neurospheres with a diameter greater than or equal to 30 μm were considered in the quantitative analysis, excluding smaller cellular aggregates (Table 2). In contrast, SZ‐derived cultures exhibited only sparse and poorly organized clusters with occasional immature neurosphere‐like structures (Figure 2B).

**Table 2:** Quantitative analysis of neurosphere formation in HC‐ and SZ‐derived ONSPCs under EGF stimulation and following EGFR inhibition.

HC	EGF (mean ± SD)	EGF (CV%)	EGF + erlotinib (mean ± SD)	EGF + inhibitor (CV%)	EGF + inhibitor (% inhibition)
1	2521 ± 214	8.5	1187 ± 185	15.6	47.1
2	3865 ± 316	8.2	1971 ± 221	11.2	51.1
3	2319 ± 234	10.1	881 ± 115	13.1	38.0
4	3513 ± 473	13.5	1932 ± 280	14.5	55.0
5	2954 ± 181	6.1	1270 ± 145	11.4	43.0
6	3001 ± 174	5.8	1560 ± 205	13.1	52.1
**HC group summary**	3029 ± 583†		1467 ± 433‡		
**SZ**					
1	321 ± 13	4.0	n.d.	n.d.	n.d.
2	454 ± 14	3.1	n.d.	n.d.	n.d.
3	246 ± 57	23.2	n.d.	n.d.	n.d.
4	587 ± 16	2.7	n.d.	n.d.	n.d.
5	811 ± 13	1.6	n.d.	n.d.	n.d.
6	439 ± 80	18.2	n.d.	n.d.	n.d.
**SZ group summary**	476 ± 202†				

*Note:* Values represent donor‐level means ± SD derived from three technical replicates for each experimental condition. CV%, coefficient of variation across technical replicates. Group summaries were calculated from donor means (*n* = 6 biological replicates per group). Between‐group comparisons (HC vs SZ under EGF conditions) were performed using the two‐sided Mann–Whitney *U* test. Comparisons before and after erlotinib treatment were performed using the Wilcoxon signed‐rank test. Percentage inhibition was calculated individually for each donor as the relative reduction in neurosphere number following EGFR inhibition. † *p* < 0.001; ‡ *p* = 0.031 n.d., not determined.

These observations differed markedly from those obtained under complete NS‐A proliferation conditions, suggesting impaired EGFR‐dependent signaling in SZ‐derived ONSPCs. To directly assess the contribution of EGFR signaling to neurosphere formation, ONSPCs were pretreated with the selective EGFR inhibitor erlotinib (10 μM) for 30 min prior to stimulation with 20 ng/mL EGF for 48 h. Erlotinib substantially reduced neurosphere formation in HC‐derived cultures (Figure 2C), while virtually abolishing the already limited aggregative capacity of SZ‐derived ONSPCs (Figure 2D). Collectively, these findings support a critical role for EGFR signaling in sustaining neural stem/progenitor cell proliferation and neurosphere‐forming capacity.

### 3.3 SZ‐Derived ONSPCs Show Perturbed Signaling Downstream of EGF Engagement

**Figure 3:**
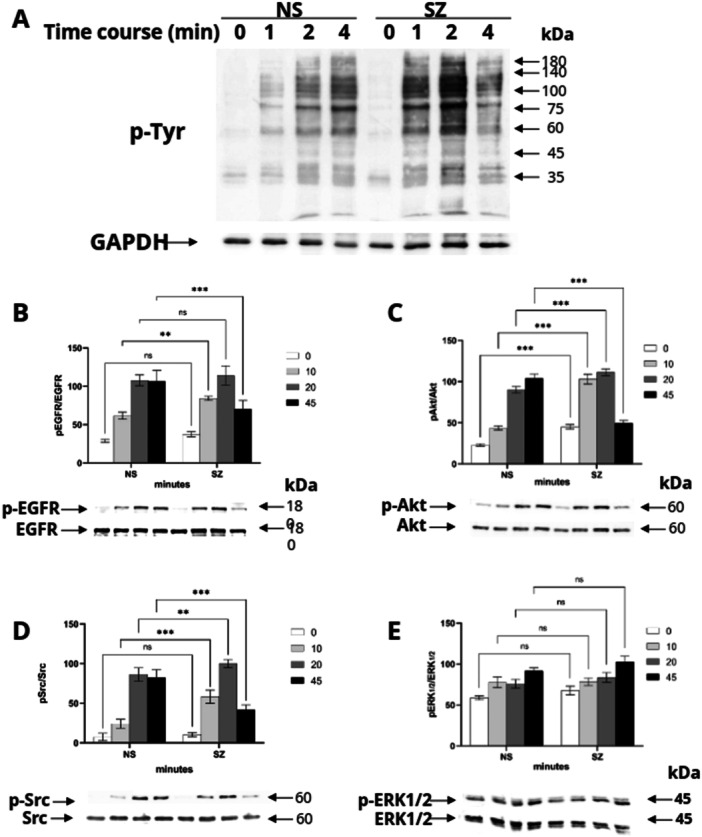
Time‐course analysis of the phosphorylation status of EGFR and downstream effectors following EGFR engagement in HC‐ and SZ‐derived ONSPCs. Cell lysates from HC‐ and SZ‐derived ONSPCs seeded into 12‐well plates (8 × 10^4^ cells per well) and treated with 20 ng/mL EGF for 0, 10, 20, 45 min were subject to WB analysis with antibodies against the phosphorylated forms of tyrosine, EGFR, Akt, Src, and ERK1/2 (A, B, C, D, and E, respectively). Band intensity was quantified by densitometric analysis of chemiluminescent signals using ImageJ software and normalized to GAPDH, used as a loading control. The normalized values are presented as bar graphs showing mean ± S.D. ns, not significant; *p < 0.05, **p < 0.01, ***p < 0.001.

**Figure 4:**
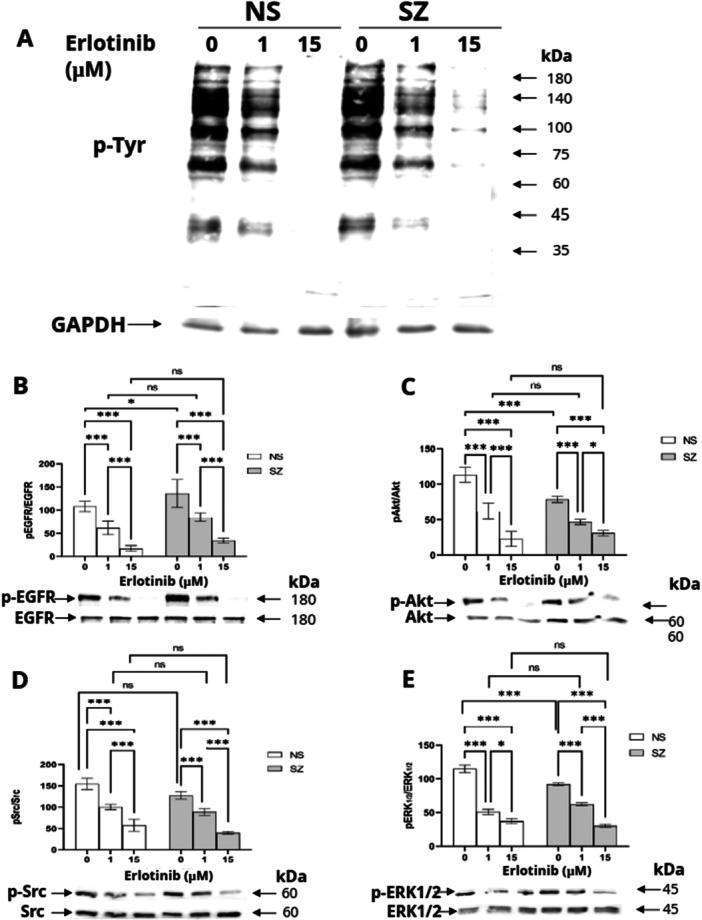
Effect of increasing concentrations of erlotinib on the phosphorylation status of EGFR and downstream effectors following EGFR engagement in HC‐ and SZ‐derived ONSPCs. Cell lysates from HC‐ and SZ‐derived ONSPCs seeded into 12‐well plates (8 × 10^4^ cells per well), pretreated with increasing concentrations of erlotinib (0, 1, 15 μM) for 30 min and supplemented with 20 ng/mL EGF for 20 min, were subject to WB analysis with antibodies against the phosphorylated forms of tyrosine, EGFR, Akt, Src, and ERK1/2 (A, B, C, D, and E, respectively). Band intensity was quantified by densitometric analysis of chemiluminescent signals using ImageJ software and normalized to GAPDH, used as a loading control. The normalized values are presented as bar graphs showing mean ± S.D. ns, not significant; *p < 0.05, **p < 0.01, ***p < 0.001.

To investigate the molecular mechanisms underlying the differential response to EGF between the two participant groups, we examined the EGFR signaling cascade, focusing on receptor activation and key downstream effectors, including Akt, Src, and ERK1/2 (Singh et al. 2012a; Wee and Wang 2017). ONSPCs (8 × 10^4^) were seeded in 12‐well plates and cultured for 24 h in complete DMEM/F12 at 37°C under 5% CO_2_ to reach ~80% confluence. WB analysis using anti‐EGFR antibodies revealed comparable total EGFR expression levels in both groups (Figure 3B). To assess EGF‐induced signaling dynamics, ONSPCs were serum‐starved for 24 h and stimulated with 20 ng/mL EGF for 0, 10, 20, and 45 min. WB analysis with anti‐phosphotyrosine antibodies revealed a global phosphotyrosine profile that differed markedly between groups, with HC‐derived ONSPCs displaying a progressive and sustained increase in tyrosine phosphorylation, whereas SZ‐derived ONSPCs exhibited an earlier but transient phosphotyrosine signal (Figure 3A). At the receptor level, HC‐derived ONSPCs showed modest EGFR phosphorylation that increased progressively, reaching a peak at 20 min and plateauing thereafter. In contrast, SZ‐derived ONSPCs exhibited rapid and pronounced EGFR phosphorylation detectable as early as 10 min, peaking at 20 min but declining markedly by 45 min (Figure 3B). Phosphorylation of Akt followed a pattern closely mirroring that of EGFR in both groups. HC‐derived ONSPCs showed gradual and sustained Akt activation, whereas SZ‐derived ONSPCs displayed earlier but transient phosphorylation followed by a pronounced decline at 45 min (Figure 3C). A comparable pattern was observed for Src, with HC‐derived ONSPCs exhibiting progressive phosphorylation and SZ‐derived ONSPCs showing earlier yet rapidly attenuated activation (Figure 3D). By contrast, ERK1/2 phosphorylation kinetics were comparable between groups, indicating no differential engagement of the MAPK branch (Figure 3E). To verify pathway specificity, ONSPCs were preincubated with increasing concentrations of erlotinib (0, 1, and 15 μM) for 30 min prior to stimulation with EGF for 30 min. Global tyrosine phosphorylation, assessed by WB analysis with anti‐phosphotyrosine antibodies, was markedly reduced by erlotinib in a dose‐dependent manner in both HC‐ and SZ‐derived ONSPCs, with near‐complete abrogation at the highest concentration, confirming on‐target engagement of the inhibitor (Figure 4A). Indeed, erlotinib abolished EGFR phosphorylation in a dose‐dependent manner (Figure 4B), with parallel suppression of downstream Akt, Src, and ERK1/2 activation (Figure 4C,D,E, respectively). Collectively, these findings suggest that the aberrant magnitude and temporal dynamics of EGF‐driven signaling observed in SZ‐derived ONSPCs reflect dysregulation at the receptor level, consistent with a direct contribution of aberrant EGFR activity to the impaired neurosphere‐forming capacity of these cells.

## 4 Discussion

Schizophrenia affects approximately 1% of the global population, imposing considerable personal and societal burdens. Despite advances in elucidating its etiopathogenesis, the underlying biological mechanisms of schizophrenia remain incompletely understood. Although conventional experimental models—including post‐mortem brain tissue, animal models, and induced pluripotent stem cells—have provided valuable mechanistic insights, each is constrained by inherent limitations, such as loss of patient‐specific signatures, high costs, and technical challenges (Lipska et al. 2006; Volpato and Webber 2020; Bellon 2024).

By contrast, ONSPCs represent a practical and physiologically relevant human‐based alternative. Crucially, they closely recapitulate mid‐fetal brain progenitor properties at a developmental window during which environmental insults may increase vulnerability to psychiatric disorders (Tebbenkamp et al. 2014), rendering them particularly suitable for exploring schizophrenia within the neurodevelopmental paradigm (Khandaker et al. 2013). ONSPCs retain neural commitment and preserve epigenetic features that reflect donor identity and disease status, while established culture and cryopreservation protocols support efficient expansion and longitudinal analyses (Borgmann‐Winter et al. 2015; Idotta et al. 2024). Their anatomical accessibility further enables straightforward collection — most commonly by nasal biopsy (Vitale et al. 2017), or alternatively by a non‐invasive, low‐cost brushing technique that minimizes discomfort and broadens applicability (Benítez‐King et al. 2011; Idotta et al. 2024).

In line with previous studies, whereby we identified functional alterations in SZ‐derived ONSPCs consistent with findings from other experimental models (Idotta et al. 2019; Idotta et al. 2024), we focused on signaling pathways downstream of EGF engagement, a key regulator of neurodevelopment. Specifically, we investigated whether perturbations in EGF signaling are associated with morpho‐functional abnormalities that distinguish SZ‐derived ONSPCs from neurotypical controls.

Our findings provide evidence that SZ‐derived ONSPCs display distinct functional and signaling responses to EGF compared to HCs. Both groups formed neurospheres, a standard readout of neural stem cell self‐renewal and proliferation, under proliferative conditions in the presence of EGF and FGF (Reynolds and Weiss 1992), critical growth factors in neurodevelopment, but when exposed to EGF‐exclusive conditions, HC‐derived ONSPCs formed typical neurospheres, whereas the SZ‐derived counterparts generated sparse, disorganized aggregates. This deficit occurred despite comparable EGFR protein levels, suggesting that altered signaling dynamics rather than receptor abundance underlie impaired responsiveness. This finding is consistent with a broader pattern of proliferative abnormalities in patient‐derived neural cells, and extends previous evidence from our group documenting impaired proliferation in SZ‐derived ONSPCs relative to HCs, particularly with increasing passage number (Idotta et al. 2019) and following cryopreservation and thawing (Idotta et al. 2024). These observations may appear to contrast with earlier seminal studies reporting enhanced proliferative activity in SZ‐derived olfactory neuroepithelium, as evidenced by elevated mitotic index (Féron et al. 1999), dysregulated expression of cell‐cycle regulatory genes (McCurdy et al. 2006), and increased expression of key drivers of cell‐cycle progression—cyclins D1, E, and A2—consistent with a proliferative bias (Fan et al. 2012). However, this apparent discrepancy is likely attributable to differences in experimental conditions: those studies were conducted either on tissue sections and cultures supplemented with FGF (Féron et al. 1999; McCurdy et al. 2006), or on thawed cells plated on extracellular matrix‐mimicking substrates such as fibronectin (Fan et al. 2012), whereas stringent EGF‐exclusive conditions selectively unmask impaired neurosphere formation and aberrant EGFR‐dependent signaling in SZ‐derived cells. Whether this reflects a genuine pathophysiological signature remains unclear and will require further investigation. In particular, experimental paradigms incorporating adhesion substrates and FGF‐exclusive conditions may help define the respective roles and potential overlap of EGFR‐ and FGFR‐dependent signaling in this model.

In this context, aberrant EGFR–Akt signaling dynamics represent a candidate upstream mechanism through which cell‐cycle entry and neurosphere‐forming capacity may be disrupted in SZ‐derived ONSPCs, given the well‐established role of PI3K/Akt signaling in G1/S transition (Sheng et al. 2003) and progenitor self‐renewal (Singh et al. 2012b).

Indeed, SZ‐derived ONSPCs exhibited earlier and stronger, yet transient, EGFR activation, accompanied by rapid phosphorylation and decay of downstream effectors, particularly Akt and Src, whereas HC‐derived ONSPCs displayed weaker but sustained activation over time. These findings suggest that premature attenuation of EGFR–PI3K/Akt signaling in SZ‐derived ONSPCs may contribute to reduced proliferative responsiveness and impaired neurosphere formation, consistent with altered regulation of neural stem/progenitor cell self‐renewal. The sensitivity of both signaling and neurosphere formation to pharmacological EGFR inhibition further supports a central role for EGFR‐dependent pathways in sustaining neural progenitor function (Le Belle et al. 2011). By contrast, ERK1/2 activation within the EGFR–MAPK branch appeared comparatively preserved in both groups, in line with the relative preservation of differentiation‐related signaling and further supporting the hypothesis of preferential vulnerability of the EGFR–PI3K/Akt axis in SZ‐derived ONSPCs.

Our findings are consistent with broader evidence linking dysregulated EGF/ErbB signaling to schizophrenia. Post‐mortem data show reduced EGF and increased EGFR in the patient prefrontal cortex (Futamura et al. 2002), while rodent studies reveal that perinatal EGF exposure induces schizophrenia‐like behaviors reversible by EGFR inhibition (Mizuno et al. 2013). These observations suggest that aberrant temporal regulation of EGFR–Akt signaling in neural progenitors may contribute to the developmental disruptions underlying schizophrenia.

Beyond the potentially relevant findings of the present study, several limitations should be acknowledged. The relatively small cohort size limits statistical power and may reduce the generalizability of the results. Moreover, all patients with schizophrenia were receiving antipsychotic treatment at the time of sampling, and therefore, a potential influence of medication exposure on EGFR signaling dynamics and neurosphere‐forming capacity cannot be excluded, although repeated culture passages may reduce direct pharmacological effects. In this regard, studies in medication‐naïve individuals would be informative. Regarding the expression and phosphorylation levels of the signaling proteins under investigation, approaches yielding absolute abundance measures—such as mass spectrometry‐based phosphoproteomics—would go beyond the semiquantitative nature of Western blot analysis, albeit mitigated by normalization to housekeeping proteins, and could further corroborate the present findings. In addition, laboratory analyses were not performed blind to participant group status, potentially introducing observer bias. Future studies involving larger cohorts, medication‐naïve patients, blinded analyses, and complementary quantitative approaches will be necessary to validate and extend the present findings.

In summary, SZ‐derived ONSPCs display rapid but unsustained EGFR and Akt activation, early Src engagement, and preserved ERK1/2 signaling. This atypical signaling pattern is associated with defective neurosphere formation under EGF‐exclusive conditions, suggesting altered regulation of stemness‐related signaling and self‐renewal capacity. Future studies should test whether prolonging Akt activation can restore these deficits, providing mechanistic insight and potential therapeutic avenues for neurodevelopmental vulnerability in schizophrenia.

## Author Contributions

Study conceptualization, design, supervision, formal analysis, original draft, manuscript review, and editing: Tommaso Toffanin, Mario Angelo Pagano. Biochemical and microscopy analyses: Mauro Salvi, Valentina Bosello‐Travain, Maria Lina Massimino, and Anna Maria Brunati. Sample collection: Roberto Saetti and Marina Silvestrini. Patient screening, diagnostic assessments, and classified patients according to the inclusion criteria: Leonardo Meneghetti, Stefano Piazza, Maria Giulia Nanni, Rosangela Caruso, Maria Ferrara, Chiara Montemitro, Luigi Zerbinati, Martino Belvederi Murri. Supervision, funding acquisition, manuscript review, and editing: Carlo Idotta and Luigi Grassi.

## Ethics Statement

This study was conducted in compliance with the ethical principles outlined in the Declaration of Helsinki and was approved by the Vicenza Ethical Review Committee (Protocol No. 1022/23). All participants provided written informed consent prior to enrollment. Informed consent was obtained from all participants in the study.

## Conflicts of Interest

The authors declare no conflicts of interest.

## Data Availability

The data that support the findings of this study are available from the corresponding author upon reasonable request.

## References

[cbin70181-bib-0001] Aguirre, A. , M. E. Rubio , and V. Gallo . 2010. “Notch and EGFR Pathway Interaction Regulates Neural Stem Cell Number and Self‐Renewal.” Nature 467: 323–327. 10.1038/nature09347. 20844536 PMC2941915

[cbin70181-bib-0002] Bellon, A. 2024. “Comparing Stem Cells, Transdifferentiation and Brain Organoids as Tools for Psychiatric Research.” Translational Psychiatry 14, no. 14: 127. 10.1038/s41398-024-02780-8. 38418498 PMC10901833

[cbin70181-bib-0003] Benítez‐King, G. , A. Riquelme , L. Ortíz‐López , et al. 2011. “A Non‐Invasive Method to Isolate the Neuronal Linage From the Nasal Epithelium From Schizophrenic and Bipolar Diseases.” Journal of Neuroscience Methods 201, no. 1: 35–45. 10.1016/j.jneumeth.2011.07.009. 21787803

[cbin70181-bib-0004] Borgmann‐Winter, K. , S. L. Willard , D. Sinclair , et al. 2015. “Translational Potential of Olfactory Mucosa for the Study of Neuropsychiatric Illness.” Translational Psychiatry 5, no. 3: e527. 10.1038/tp.2014.141. 25781226 PMC4354342

[cbin70181-bib-0005] Burrows, R. C. , D. Wancio , P. Levitt , and L. Lillien . 1997. “Response Diversity and the Timing of Progenitor Cell Maturation Are Regulated by Developmental Changes in EGFR Expression in the Cortex.” Neuron 19, no. 2: 251–267. 10.1016/S0896-6273(00)80937-X. 9292717

[cbin70181-bib-0006] Chen, J. , F. Zeng , S. J. Forrester , S. Eguchi , M.‐Z. Zhang , and R. C. Harris . 2016. “Expression and Function of the Epidermal Growth Factor Receptor in Physiology and Disease.” Physiological Reviews 96, no. 3: 1025–1069. 10.1152/physrev.00030.2015. 33003261

[cbin70181-bib-0007] da Silva Siqueira, L. , F. Majolo , A. P. B. da Silva , J. C. da Costa , and D. R. Marinowic . 2021. “Neurospheres: A Potential In Vitro Model for the Study of Central Nervous System Disorders.” Molecular Biology Reports 48, no. 4: 3649–3663. 10.1007/s11033-021-06301-4. 33765252

[cbin70181-bib-0008] Doetsch, F. , L. Petreanu , I. Caille , J.‐M. Garcia‐Verdugo , and A. Alvarez‐Buylla . 2002. “EGF Converts Transit‐Amplifying Neurogenic Precursors in the Adult Brain Into Multipotent Stem Cells.” Neuron 36, no. 6: 1021–1034. 10.1016/S0896-6273(02)01133-9. 12495619

[cbin70181-bib-0009] Ezaddoustdar, A. , D. Kalina , M. Bielohuby , M. Boehm , and M. Wygrecka . 2025. “Deregulated Pathways: Unraveling the Role of Epiregulin in Skin, Kidney, and Lung Fibrosis.” American Journal of Physiology ‐ Cell Physiology 328, no. 2: C617–C626. 10.1152/ajpcell.00813.2024. 39750963

[cbin70181-bib-0010] Fan, Y. , G. Abrahamsen , J. J. McGrath , and A. Mackay‐Sim . 2012. “Altered Cell Cycle Dynamics in Schizophrenia.” Biological Psychiatry 71, no. 2: 129–135. 10.1016/j.biopsych.2011.10.004. 22074612

[cbin70181-bib-0011] Féron, F. , C. Perry , M. H. Hirning , J. McGrath , and A. Mackay‐Sim . 1999. “Altered Adhesion, Proliferation and Death in Neural Cultures From Adults With Schizophrenia.” Schizophrenia Research 40, no. 3: 211–218. 10.1016/s0920-9964(99)00055-9. 10638859

[cbin70181-bib-0012] Futamura, T. , K. Toyooka , S. Iritani , et al. 2002. “Abnormal Expression of Epidermal Growth Factor and Its Receptor in the Forebrain and Serum of Schizophrenic Patients.” Molecular Psychiatry 7, no. 7: 673–682. 10.1038/sj.mp.4001081. 12192610

[cbin70181-bib-0013] Gao, C. , W. Wang , T. Liu , X. Li , Y. Yu , and J. Wu . 2025. “Annual Review of EGFR Inhibitors in 2024.” European Journal of Medicinal Chemistry 292: 117677. 10.1016/j.ejmech.2025.117677. 40328037

[cbin70181-bib-0014] Hu, Q. , L. Zhang , J. Wen , et al. 2010. “The Egf Receptor‐Sox2‐Egf Receptor Feedback Loop Positively Regulates the Self‐Renewal of Neural Precursor Cells.” Stem Cells 28, no. 2: 279–286. 10.1002/stem.246. 19882665

[cbin70181-bib-0015] Idotta, C. , E. Tibaldi , A. M. Brunati , et al. 2019. “Olfactory Neuroepithelium Alterations and Cognitive Correlates in Schizophrenia.” European Psychiatry 61: 23–32. 10.1016/j.eurpsy.2019.06.004. 31260908

[cbin70181-bib-0016] Idotta, C. , M. A. Pagano , E. Tibaldi , et al. 2024. “Neural Stem/Progenitor Cells From Olfactory Neuroepithelium Collected by Nasal Brushing as a Cell Model Reflecting Molecular and Cellular Dysfunctions in Schizophrenia.” World Journal of Biological Psychiatry 25, no. 6: 317–329. 10.1080/15622975.2024.2357096. 38869228

[cbin70181-bib-0017] Ilaria Decimo, I. , I. Francesco Bifari , I. Mauro Krampera , and I. Guido Fumagalli . 2012. “Neural Stem Cell Niches in Health and Diseases.” Current Pharmaceutical Design 18, no. 13: 1755–1783. 10.2174/138161212799859611. 22394166 PMC3343380

[cbin70181-bib-0018] Khandaker, G. M. , J. Zimbron , G. Lewis , and P. B. Jones . 2013. “Prenatal Maternal Infection, Neurodevelopment and Adult Schizophrenia: A Systematic Review of Population‐Based Studies.” Psychological Medicine 43, no. 2: 239–257. 10.1017/S0033291712000736. 22717193 PMC3479084

[cbin70181-bib-0019] Le Belle, J. E. , N. M. Orozco , A. A. Paucar , et al. 2011. “Proliferative Neural Stem Cells Have High Endogenous ROS Levels That Regulate Self‐Renewal and Neurogenesis in a PI3K/Akt‐Dependant Manner.” Cell Stem Cell 8, no. 1: 59–71. 10.1016/j.stem.2010.11.028. 21211782 PMC3018289

[cbin70181-bib-0020] Lemmon, M. A. , and J. Schlessinger . 2010. “Cell Signaling by Receptor Tyrosine Kinases.” Cell 141, no. 7: 1117–1134. 10.1016/j.cell.2010.06.011. 20602996 PMC2914105

[cbin70181-bib-0021] Lemmon, M. A. , J. Schlessinger , and K. M. Ferguson . 2014. “The EGFR Family: Not So Prototypical Receptor Tyrosine Kinases.” Cold Spring Harbor Perspectives in Biology 6, no. 4: a020768. 10.1101/cshperspect.a020768. 24691965 PMC3970421

[cbin70181-bib-0022] Levantini, E. , G. Maroni , M. Del Re , and D. G. Tenen . 2022. “EGFR Signaling Pathway as Therapeutic Target in Human Cancers.” Seminars in Cancer Biology 85: 253–275. 10.1016/j.semcancer.2022.04.002. 35427766

[cbin70181-bib-0023] Lillien, L. , and H. Raphael . 2000. “BMP and FGF Regulate the Development of EGF‐Responsive Neural Progenitor Cells.” Development 127, no. 22: 4993–5005. 10.1242/dev.127.22.4993. 11044412

[cbin70181-bib-0024] Lipska, B. K. , A. Deep‐Soboslay , C. S. Weickert , et al. 2006. “Critical Factors in Gene Expression in Postmortem Human Brain: Focus on Studies in Schizophrenia.” Biological Psychiatry 60, no. 6: 650–658. 10.1016/j.biopsych.2006.06.019. 16997002

[cbin70181-bib-0025] Mackay‐Sim, A. 2012. “Concise Review: Patient‐Derived Olfactory Stem Cells: New Models for Brain Diseases.” Stem Cells 30, no. 11: 2361–2365. 10.1002/stem.1220. 22961669

[cbin70181-bib-0026] McCurdy, R. D. , F. Féron , C. Perry , et al. 2006. “Cell Cycle Alterations in Biopsied Olfactory Neuroepithelium in Schizophrenia and Bipolar I Disorder Using Cell Culture and Gene Expression Analyses.” Schizophrenia Research 82, no. 2–3: 163–173. 10.1016/j.schres.2005.10.012. 16406496

[cbin70181-bib-0027] Mizuno, M. , H. Sotoyama , H. Namba , et al. 2013. “ErbB Inhibitors Ameliorate Behavioral Impairments of an Animal Model for Schizophrenia: Implication of Their Dopamine‐Modulatory Actions.” Translational Psychiatry 3, no. 4: e252. 10.1038/tp.2013.29. 23632456 PMC3641415

[cbin70181-bib-0028] Namba, H. , and H. Nawa . 2020. “Post‐Pubertal Difference in Nigral Dopaminergic Cells Firing in the Schizophrenia Model Prepared by Perinatal Challenges of a Cytokine, EGF.” Neuroscience 441: 22–32. 10.1016/j.neuroscience.2020.06.003. 32531471

[cbin70181-bib-0029] Pantazopoulos, H. , P. Katsel , V. Haroutunian , G. Chelini , T. Klengel , and S. Berretta . 2021. “Molecular Signature of Extracellular Matrix Pathology in Schizophrenia.” European Journal of Neuroscience 53, no. 12: 3960–3987. 10.1111/ejn.15009. 33070392 PMC8359380

[cbin70181-bib-0030] Pastore, S. , F. Mascia , V. Mariani , and G. Girolomoni . 2008. “The Epidermal Growth Factor Receptor System in Skin Repair and Inflammation.” Journal of Investigative Dermatology 128, no. 6: 1365–1374. 10.1038/sj.jid.5701184. 18049451

[cbin70181-bib-0031] Purba, E. , E. Saita , and I. Maruyama . 2017. “Activation of the EGF Receptor by Ligand Binding and Oncogenic Mutations: The ‘Rotation Model.” Cells 6, no. 2: 13. 10.3390/cells6020013. 28574446 PMC5492017

[cbin70181-bib-0032] Rapoport, J. L. , A. M. Addington , S. Frangou , and M. R. C. Psych . 2005. “The Neurodevelopmental Model of Schizophrenia: Update 2005.” Molecular Psychiatry 10, no. 5: 434–449. 10.1038/sj.mp.4001642. 15700048

[cbin70181-bib-0033] Reynolds, B. A. , and S. Weiss . 1992. “Generation of Neurons and Astrocytes From Isolated Cells of the Adult Mammalian Central Nervous System.” Science 255, no. 5052: 1707–1710. 10.1126/science.1553558. 1553558

[cbin70181-bib-0034] Sheng, H. , J. Shao , C. M. Townsend , and B. M. Evers . 2003. “Phosphatidylinositol 3‐kinase Mediates Proliferative Signals in Intestinal Epithelial Cells.” Gut 52, no. 10: 1472–1478. 10.1136/gut.52.10.1472. 12970141 PMC1773820

[cbin70181-bib-0035] Sibilia, M. , and E. F. Wagner . 1995. “Strain‐Dependent Epithelial Defects in Mice Lacking the EGF Receptor.” Science 269, no. 5221: 234–238. 10.1126/science.7618085. 7618085

[cbin70181-bib-0036] Singh, A. M. , D. Reynolds , T. Cliff , et al. 2012b. “Signaling Network Crosstalk in Human Pluripotent Cells: A Smad2/3‐regulated Switch That Controls the Balance Between Self‐Renewal and Differentiation.” Cell Stem Cell 10, no. 3: 312–326. 10.1016/j.stem.2012.01.014. 22385658 PMC3294294

[cbin70181-bib-0037] Singh, S. , J. Trevino , N. Bora‐Singhal , et al. 2012a. “EGFR/SRC/Akt Signaling Modulates Sox2 Expression and Self‐Renewal of Stem‐Like Side‐Population Cells in Non‐Small Cell Lung Cancer.” Molecular Cancer 11, no. 1: 73. 10.1186/1476-4598-11-73. 23009336 PMC3497614

[cbin70181-bib-0038] Sun, Y. , S. K. Goderie , and S. Temple . 2005. “Asymmetric Distribution of EGFR Receptor During Mitosis Generates Diverse CNS Progenitor Cells.” Neuron 45, no. 6: 873–886. 10.1016/j.neuron.2005.01.045. 15797549

[cbin70181-bib-0039] Taylor, D. M. , T. R. E. Barnes , and A. H. Young . 2025. The Maudsley Prescribing Guidelines in Psychiatry 15th ed. John Wiley & Sons.

[cbin70181-bib-0040] Tebbenkamp, A. T. N. , A. J. Willsey , M. W. State , and N. Šestan . 2014. “The Developmental Transcriptome of the Human Brain: Implications for Neurodevelopmental Disorders.” Current Opinion in Neurology 27, no. 2: 149–156. 10.1097/WCO.0000000000000069. 24565942 PMC4038354

[cbin70181-bib-0041] Vitale, A. M. , N. A. Matigian , A. S. Cristino , et al. 2017. “DNA Methylation in Schizophrenia in Different Patient‐Derived Cell Types.” NPJ Schizophrenia 3, no. 1: 6. 10.1038/s41537-016-0006-0. 28560252 PMC5441549

[cbin70181-bib-0042] Volpato, V. , and C. Webber . 2020. “Addressing Variability in iPSC‐Derived Models of Human Disease: Guidelines to Promote Reproducibility.” Disease Models & Mechanisms 13, no. 1: dmm042317. 10.1242/dmm.042317. 31953356 PMC6994963

[cbin70181-bib-0043] Wagner, B. , A. Natarajan , S. Grünaug , R. Kroismayr , E. F. Wagner , and M. Sibilia . 2006. “Neuronal Survival Depends on EGFR Signaling in Cortical but Not Midbrain Astrocytes.” EMBO Journal 25, no. 4: 752–762. 10.1038/sj.emboj.7600988. 16467848 PMC1383568

[cbin70181-bib-0044] Wee, P. , and Z. Wang . 2017. “Epidermal Growth Factor Receptor Cell Proliferation Signaling Pathways.” Cancers 9, no. 5: 52. 10.3390/cancers9050052. 28513565 PMC5447962

[cbin70181-bib-0045] Yu, S.‐J. , H.‐J. Kim , E. S. Lee , C.‐G. Park , S. J. Cho , and S.‐H. Jeon . 2017. “β‐Catenin Accumulation is Associated With Increased Expression of Nanog Protein and Predicts Maintenance of MSC Self‐Renewal.” Cell Transplantation 26, no. 2: 365–377. 10.3727/096368916X693040. 27684957 PMC5657765

